# Hiatal Hernia: A Possible Trigger for Atrial Fibrillation

**DOI:** 10.7759/cureus.18857

**Published:** 2021-10-18

**Authors:** Atika Malik, Karimah Best, Sahiljeet Singh, Kory S Jaggon, Miriam Michael

**Affiliations:** 1 Internal Medicine, Punjab Hospital, Sialkot, PAK; 2 Internal Medicine, American University of Antigua, Osbourn, ATG; 3 Internal Medicine, University of Maryland, Baltimore, USA

**Keywords:** gastrocardiac syndrome, hiatal hernia, arrhythmia, atrial fibrillation, nissen fundoplication

## Abstract

Atrial fibrillation is a commonly encountered clinical entity with various cardiovascular consequences. Common risk factors include alcohol abuse, hyperthyroidism, mitral stenosis, hypertension, diabetes mellitus, and coronary artery disease. Another risk factor, yet under scientific scrutiny, is hiatal hernia. This anatomical abnormality, due to its proximity to the heart and high prevalence in atrial fibrillation patients, has merited scientific investigation to determine if an association truly exists between this gastrointestinal pathology and atrial fibrillation. The case herein is of an 81-year-old hospitalized female with a hiatal hernia who was recorded to have recurrent episodes of atrial fibrillation in the absence of traditional risk factors for arrhythmogenesis.

## Introduction

Atrial fibrillation is the most commonly encountered cardiac arrhythmia [1]. It arises due to abnormal electrical activity within the pulmonary veins which leads to unsynchronized contraction of the atria and hence, cardiovascular compromise. It is broadly classified into two major categories, valvular atrial fibrillation and non-valvular atrial fibrillation, wherein the pathophysiology of both revolves around atrial remodeling. Mitral stenosis is the most common risk factor for valvular atrial fibrillation [2]. The most common risk factors for non-valvular atrial fibrillation include alcohol abuse, hyperthyroidism, hypertension, diabetes mellitus, cardiomyopathy, and coronary artery disease [3-5]. A relatively less explored and plausible risk factor for atrial fibrillation is hiatal hernia [6], whereby a herniated stomach causes direct mechanical compression of the atria leading to arrhythmogenesis. Here, we present the case of an 81-year-old hospitalized female with a hiatal hernia who experienced repeated episodes of atrial fibrillation in the absence of traditional risk factors.

## Case presentation

**Figure 1:**
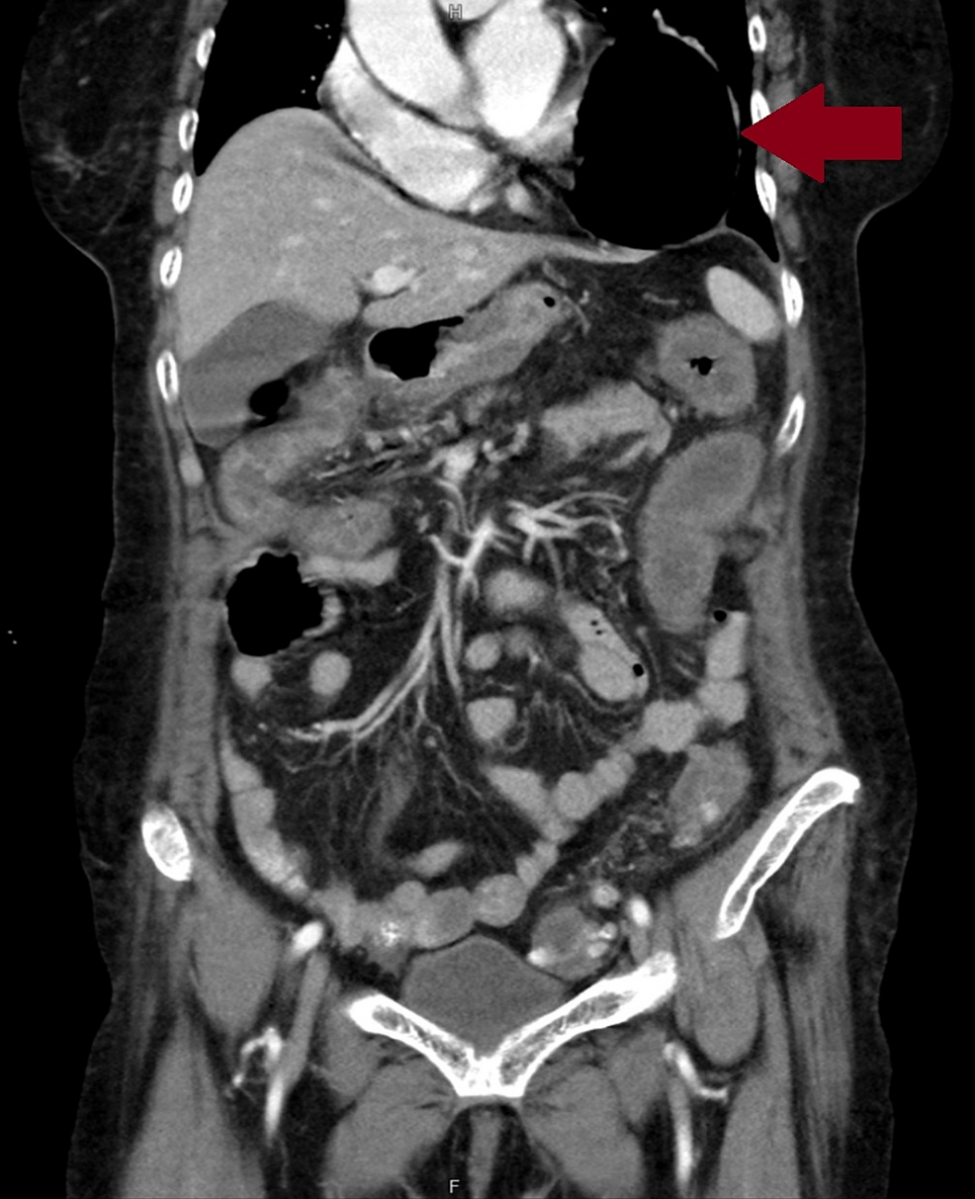
CT scan The red arrow points to the hiatal hernia compressing the heart

An 81-year-old Chinese female presented to the emergency room with a one-day history of bloody diarrhea and generalized abdominal pain. She denied hematemesis or other associated symptoms. Past medical history was significant for osteoarthritis, hyperparathyroidism, hypothyroidism, iron deficiency anemia, and anxiety disorder. A review of systems was unremarkable apart from the aforementioned complaints. On physical examination, vital signs were recorded as heart rate (HR): 98 beats/minute (bpm), blood pressure (BP): 100/70 mmHg, temperature: 98 °F, respiratory rate (RR): 17 breaths/minute. Her body mass index (BMI) was 25.5 kg/m². The abdomen was tender in the right lower quadrant (RLQ), left lower quadrant (LLQ), and suprapubic area. Laboratory testing showed elevated WBC count (11,500/microlitre), increased blood urea nitrogen (BUN) (35 mg/dL), hypermagnesemia (2.9 mg/dL), hypercalcemia (14.2 mg/dL), and hyperglycemia (123 mg/dL). Computed tomography revealed evidence of diverticulosis and pancolitis. Of note, a large hiatal hernia was also observed (Figure 1). 

She was started on intravenous (IV) fluids, IV protonix, and lasix. Her home medications (cinacalcet, levothyroxine, ferrous sulfate, alprazolam, citalopram) were continued. Her bowel was prepped with GoLYTELY® 4L (polyethylene glycol electrolyte solution) and she was advised to take clear liquids. Additional laboratory tests (stool sample for culture, white blood cell count, calprotectin, *Clostridium difficile *antigen) were ordered. Later that day, she experienced an episode of asymptomatic atrial fibrillation with a rapid ventricular response (RVR) which was managed adequately with metoprolol (2.5 mg IV stat followed by 12.5 mg twice daily). Laboratory tests also revealed a drop in hemoglobin (Hb) (14 g/dL to 10 g/dL) for which a packed RBC transfusion was carried out the next day. Later that evening, the patient complained of anxiety. She was noted to have sinus tachycardia (HR: 120bpm) and was given alprazolam. A few hours later her HR increased again (HR: 160 bpm). She was then started on metoprolol 2.5 mg IV stat which normalized her heart rate. The next day, yet another episode of spontaneously resolving asymptomatic atrial fibrillation was noted. Cardiology consultation was requested. Echocardiography was performed which revealed normal heart structure and function. Her condition remained stable over the next day. Hence, she was cleared for discharge with advice to follow up for outpatient endoscopy.

## Discussion

Both atrial fibrillation and hiatal hernia are commonly encountered clinical entities [7,8]. They have been linked together by the term gastrocardiac syndrome. This term was first coined by Ludwig Roemheld wherein he described how certain gastrointestinal pathologies can present with cardiovascular symptoms [9]. Hiatal hernia tends to be one of these postulated gastrointestinal pathologies and is suspected to be a trigger for cardiac arrhythmias [6]. The pathophysiological basis for this assumption lies in the knowledge of the anatomy of the esophagus in relation to the heart [6]. A herniated stomach comes to lie directly in contact with the left atrium of the heart. This causes cardiac compression with the potential of arrhythmogenesis. Substantial supportive evidence of such a relationship is elucidated by the fact that with data collected over a span of thirty years during a study carried out by Mayo clinic it was observed that hiatal hernia was particularly associated with atrial fibrillation in young adults [6], a patient population in which the traditional risk factors of atrial fibrillation are usually absent. A similar finding was also observed in our patient, who experienced recurrent episodes of atrial fibrillation in the absence of traditional risk factors such as mitral stenosis, cardiomyopathy, coronary artery disease, etc. Hence, it was hypothesized that it was in fact the impingement of the hernia on the left atrium that was responsible for the arrhythmic episodes.

The probable causal relationship between hiatal hernia and atrial fibrillation is particularly important from the standpoint of management. Hiatal hernia is an easily treatable condition amenable to surgical correction. Hence, if a definitive relationship is established, these patients will be able to receive more focused management for their pathology. As a matter of fact, various studies in the past have reported a reduction in the frequency of atrial fibrillation following Nissen fundoplication [10-12]. Although existing literature does support gastrocardiac syndrome, more scientific efforts need to be directed towards this clinical entity in order to establish the presence of a direct causal relationship.

## Conclusions

From a pathophysiologic standpoint, hiatal hernia is a plausible etiology of cardiac arrhythmias. This is further supported by the fact that patients with hiatal hernias who also suffer from atrial fibrillation, if subjected to surgical correction of the defect, report a reduction in the frequency of episodes of atrial fibrillation. However, further scientific investigation is required to confirm the relationship between hiatal hernia and cardiac arrhythmias. This would ultimately assist in planning timely surgical intervention for these patients, in order to save them from the aftermath of cardiac arrhythmias.
